# Circulating Exosomal miRNAs as Biomarkers for the Diagnosis and Prognosis of Colorectal Cancer

**DOI:** 10.3390/ijms22010346

**Published:** 2020-12-31

**Authors:** Katiusse Alves dos Santos, Isabelle Cristina Clemente dos Santos, Carollyne Santos Silva, Hériks Gomes Ribeiro, Igor de Farias Domingos, Vivian Nogueira Silbiger

**Affiliations:** 1 Postgraduate Program in Pharmaceutical Sciences, Federal University of Rio Grande do Norte, RN 59012-570 Natal, Brazil; katiusse_santos@hotmail.com (K.A.d.S.); isinhaccs@hotmail.com (I.C.C.d.S.); domingos_if@hotmail.com (I.d.F.D.); 2 Bioanalysis and Molecular Biotechnology Laboratory, Federal University of Rio Grande do Norte, RN 59012-570 Natal, Brazil; carollyne-santos@hotmail.com (C.S.S.); heriksgomes@gmail.com (H.G.R.); 3 Department of Clinical and Toxicological, Federal University of Rio Grande do Norte, RN 59012-570 Natal, Brazil

**Keywords:** colorectal cancer, biomarkers, exosomal, miRNAs, circulating

## Abstract

Colorectal cancer (CRC) is one of the most common malignant tumors in the gastrointestinal tract. It is a multifactorial disease that involves environmental factors, genetic factors, and lifestyle factors. Due to the absence of specific and sensitive biomarkers, CRC patients are usually diagnosed at an advanced stage and consequently suffer from a low 5-year overall survival rate. Despite improvements in surgical resection and adjuvant chemotherapy, the prognosis of patients with CRC remains unfavorable due to local and distant metastases. Several studies have shown that small noncoding RNAs, such as microRNAs packed in exosomes, are potential biomarkers in various types of cancers, including CRC, and that they can be detected in a stable form in both serum and plasma. In this review, we report the potential of circulating exosomal miRNAs to act as biomarkers for the diagnosis and prognosis of CRC.

## 1. Introduction

Colorectal cancer (CRC) is the third most common cancer in men and the second in women. The latest data from the Global Cancer Observatory estimated over 1.8 million new cases of CRC and more than 880,000 deaths in 2018 [1]. Excessive alcohol consumption, obesity, smoking, physical inactivity, age, and family history are among the risk factors for CRC, which has led to an increase in the number of cases in recent years [2]. The tests for screening and monitoring the prognosis of the disease have important limitations. Fecal occult blood test (FOBT), colonoscopy, carcinoembryonic antigen (CEA), fecal DNA test, and carbohydrate antigen 19–9 (CA19–9) have low sensitivity and specificity or a high percentage of false-positive results. Furthermore, colonoscopy is an invasive and expensive procedure [3,4,5]. Therefore, there is an urgent need to develop noninvasive techniques for the diagnosis and prognosis of this disease, such as by testing for circulating biomarkers.

The discovery of microRNAs (miRNAs) has opened a new frontier in cancer research and provides an unprecedented potential for the development of miRNA-based biomarkers that can influence downstream genes and signaling pathways [6]. miRNAs are small noncoding RNAs (approximately 20–22 nucleotides) that regulate gene expression by inhibiting translation and/or decreasing the stability of their mRNA targets [7]. They are ideal biomarkers not only for their high stability in storage and handling conditions but also for their presence in body fluids [8,9,10]. The expression of miRNAs in plasma and serum has the potential to predict patient survival, tumor stage, the presence of lymph node metastases, and the response to CRC therapy. Several studies have reported that the uncontrolled expression of miRNAs could act as potential oncogenes or suppressors in the processes of tumor development [11,12,13,14]. These characteristics have generated intense interest in the study of circulating miRNAs as diagnostic, prognostic, and predictive biomarkers for CRC.

miRNAs can be found in their free form in the bloodstream or inside small secreted extracellular vesicles with a diameter ranging from 30 to 100 nm, also called exosomes. Their primary function is the transport of molecules packed within, such as lipids, proteins, mRNA, miRNA, and long noncoding RNA, which are delivered to recipient cells that participate in the regulation of normal physiological and pathological processes of many diseases, including cancer [15]. Exosomes can be found in the blood as well as in other biological fluids, such as saliva, breast milk, urine, amniotic fluid, and semen [16,17]. Studies indicate that exosomes contain a high level of miRNAs and exosomal miRNAs that contribute to immunomodulation, chemoresistance, and metastasis in several types of tumors [18]. In this review, we present an overview of the role of exosomal miRNAs circulating in serum or plasma in patients with CRC and discuss the variability of the results, seeking to broaden the perspective of the use of miRNAs as potential noninvasive biomarkers of CRC diagnosis and prognosis.

## 2. Results

### 2.1. Exosomal miRNAs for the Screening and Diagnosis of CRC

To assess the use of circulating exosomal miRNAs as CRC biomarkers, a study was conducted in Japan in 2014. Ogata-Kawata et al. [19] analyzed serum samples from 88 patients with CRC with primary tumors, 29 patients after primary tumor resection surgery, and 11 healthy individuals. The microarray analysis identified 16 miRNAs that were significantly expressed in the exosomes of serum samples from patients with CRC compared to healthy individuals. Among these, the expression of 16 miRNAs (let-7a, miR-1224-5p, miR-1229, miR-1246, miR-150, miR-21, miR-223, and miR-23a) was significantly reduced after surgical resection of primary tumors. miR-23a and miR-1246 showed sensitivities of 95.5% and 92%, respectively, in the analysis of the receiver operating characteristic curve (ROC). Lastly, in qRT-PCR validation, seven miRNAs (let-7a, miR-1229, miR-1246, miR-150, miR-21, miR-223, and miR-23a) were significantly expressed in the serum exosomes of patients with Classification of Malignant Tumours (TNM )stage I CRC. Recently, the increase in the exosomal expression of miR-23a was also verified by Karimi et al. [2], who investigated this and 10 other exosomal miRNA expression in serum samples from 25 patients with CRC and 13 healthy individuals in an Iranian population using qRT-PCR. Interestingly, only miR-23a and miR-301 were upregulated in patients in comparison with healthy individuals and could be used to distinguish the group of patients with CRC from the normal group, with an area under the curve (AUC) of 0.89 and 0.84, respectively. In another study conducted in China, Zhu et al. [20] conducted a four-phase survey of 196 patients with CRC and 138 healthy individuals, with the objective of identifying a serum exosomal panel of miRNAs for the diagnosis of CRC. During the screening phase, the authors analyzed the expression profiles of 168 miRNAs in three pools of peripheral serum samples from 30 patients with CRC and one combined sample from 10 healthy individuals. During the training phase, they analyzed 32 differentially expressed miRNAs for confirmation using qRT-PCR in 30 patients with CRC and 30 healthy individuals. Then, they detected 14 differentially expressed miRNAs in another set of samples (136 patients with CRC and 90 healthy individuals) during the validation phase. In parallel, the diagnostic potential and effectiveness of these miRNAs for the detection of CRC were assessed in an independent cohort with serum samples from 30 patients with CRC and 10 samples from healthy individuals. They found that among the 14 miRNAs previously detected, miR-19a-3p, miR-21-5p, and miR-425-5p were significantly upregulated in patients with CRC than in healthy individuals. The expression of these three miRNAs in exosomes was analyzed by qRT-PCR in serum samples from 10 patients with CRC and 10 healthy individuals, which showed positive regulation in the exosomes of patients with CRC. In a study conducted by Ren et al. [21], serum samples from 150 patients with CRC and 90 healthy individuals from China indicated that miR-196b-5p was significantly expressed in the serum and serum exosomes of patients with CRC compared to healthy individuals, particularly in the exosomes. ROC analysis of miR-196b-5p in the serum and serum exosomes of patients with CRC showed an AUC of 0.71 and 0.88, respectively. The results were validated by qRT-PCR. To elucidate the role of miR-146a-5p (miR-146a) in exosomes secreted by colorectal cancer stem cells (CRCSCs), Cheng et al. [22] analyzed its expression in serum samples from 53 patients with CRC from China. Using exosomal markers and qRT-PCR validation, they found that the abundance of exosomal miR-146a in circulation correlated with high levels of CD66 neutrophils; in contrast, the levels of TCD8 cells infiltrating the tumors were reduced. The authors suggested that miR-146a is the main miRNA in CRCSC exosomes and can be used as a diagnostic biomarker to identify the tumor microenvironment (TME) and immunosuppression in CRCSC neoplasms.

Additionally, exosomal miRNAs in plasma samples were investigated in subsequent studies. Wang et al. [23] analyzed nine plasma exosomal miRNAs in samples from 50 patients with CRC stage I and II from China and 50 healthy individuals. Among these miRNA candidates, miR-125a-3p and miR-320c, validated by qRT-PCR, were found to be significantly upregulated in patients with CRC compared to healthy individuals, with AUC values of 0.6849 and 0.5982, respectively. The authors also confirmed that exosomal miR-125a-3p is a potential marker for screening patients with early-stage CRC when combined with another diagnostic test (CEA and miR-125-3p AUC = 0.8552). Moreover, Zhang et al. [24] analyzed seven different exosomal miRNAs in the plasma samples of 18 patients with CRC and 18 healthy individuals. Subsequent qRT-PCR analysis indicated that four miRNAs were statistically significant (miR-17-5p, miR-181a-5p, miR-18a-5p, and miR-18b-5p), suggesting their potential as noninvasive biomarkers for the diagnosis of early CRC. In another recent study, Min et al. [25] investigated the expression of exosomal miR-92b in plasma by qRT-PCR in 114 individuals (40 patients with CRC, 22 patients with adenoma, and 52 healthy individuals) from China and demonstrated that its expression was decreased in patients with CRC. Furthermore, the authors found differences in the miRNA expression levels between patients with CRC and healthy individuals (AUC = 0.793), especially in patients with TNM stage II CRC (AUC = 0.830); however, no significant difference in expression was found between the patients with CRC and adenoma (AUC = 0.631). Thus, circulating exosomal miR-92b could be considered a good biomarker for the diagnosis of early-stage CRC, although it would be unable to differentiate between CRC and adenoma. Liu et al. [26] used qRT-PCR to evaluate the diagnostic utility of miR-486-5p in plasma and found that it was upregulated in patients with CRC compared to healthy individuals in the Chinese population, with a sensitivity of 67.5% and a specificity of 77.3% (AUC = 0.713). In contrast, it showed low levels of expression in CRC tissues. The authors also analyzed 10 plasma samples from patients with pre- and postoperative CRC to determine whether miR-486-5p expression in the plasma was derived from a tumor and found that the expression levels were significantly lower after the operation. Finally, using Gene Expression Omnibus (GEO) data, the authors suggested that miR-486-5p is derived from a tumor because it is contained in secretory exosomes.

### 2.2. Exosomal miRNAs for Estimating Prognosis in Resected Patients

Circulating exosomal miRNAs may be associated with CRC staging and severity, which are considered as potential biomarkers of a poor prognosis. Takano et al. [27] analyzed the expression of serum exosomal miR-203 in 240 patients with CRC in a study conducted in Japan. The authors observed that miR-203 expression increased significantly in a TNM stage-dependent manner and that its high expression was associated with increased aggressiveness and pathological tumor progression, including lymph node metastasis, venous invasion, distant metastasis, and advanced TNM staging in patients with CRC, as validated by qRT-PCR. In another study of 77 patients with CRC and 20 healthy individuals, Yan et al. [28] analyzed the expression of 39 miRNAs encapsulated in serum exosomes using microarrays. Among them, 10 miRNAs were upregulated, while 29 miRNAs were downregulated. qRT-PCR analysis revealed that five miRNAs encapsulated in exosomes (miR-638, miR-5787, miR-8075, miR-6869-5p, and miR-548c-5p) were downregulated, and two miRNAs (miR-486-5p and miR-3180-5p) were upregulated in patients with CRC. Interestingly, a network analysis demonstrated the ability of these five miRNAs to regulate glucose metabolism in CRC. In addition, low levels of miR-638 exosomal expression have been associated with an increased risk of liver metastases and advanced TNM stages. The following year, using the same analysis methods, in a cohort of 168 patients with CRC and 20 healthy individuals, the authors observed that the levels of exosomal miR-6803-5p in the serum increased significantly in patients with CRC, mainly in the TNM stage III/IV, with liver and lymph node metastases. Likewise, high levels of expression of this miRNA were associated with lower overall survival (OS) and progressive disease (DFS) in these patients. This finding demonstrated the influence of serum exosomal miR-6803-5p on the development and progression of CRC and its potential as a diagnostic biomarker in CRC (AUC = 0.7399) [29]. The expression of exosomal miR-6869-5p in the serum of 142 patients with CRC was found to be significantly reduced compared to 40 healthy individuals from the same population. The reduced expression was also significant in patients with CRC in the TNM IV versus I/II stage, with metastases in the liver and lymph nodes, in addition to being related to a lower survival of these patients, as determined per qRT-PCR [30]. In addition, subsequent studies analyzed the potential of circulating exosomal miRNAs as noninvasive biomarkers for the progression and metastasis of patients with CRC. Fu et al. [31] analyzed the expression of 11 miRNAs in exosomes in the serum of 18 patients with CRC, 11 patients with metastasis, and 10 healthy individuals in the Chinese population. qRT-PCR analysis showed significantly higher levels of miR-17-5p (AUC = 0.897) and miR-92a-3p (AUC = 0.845) in patients with CRC (AUC = 0.841) and in patients with metastasis (AUC = 0.854), respectively. In the same year, Peng, Gu, and Yan [32] evaluated the levels of exosomal miR-548c-5p in the serum of 108 patients with CRC using qRT-PCR. In the analyses, reduced levels of exosomal miR-548c-5p were observed in the serum of patients with CRC with hepatic metastasis and TNM stages III and IV, which were independently associated with a lower OS, predicting a poor prognosis in these patients. Recently, in a study by Tang et al. [33], the exosomal levels of nine miRNAs in the serum of 34 patients with metastatic CRC and 108 individuals with non-metastatic CRC were analyzed and validated using the same analysis methods as above. The results demonstrated high levels of miR-320d expression, with an AUC of 0.633, a sensitivity of 62.0%, and a specificity of 64.7%, suggesting its potential to differentiate patients with metastatic and nonmetastatic CRC significantly. 

The expression of exosomal miR-217 and colorectal neoplasia differentially expressed (CRNDE-p) was analyzed in the serum containing circulating exosomes of 411 patients with CRC, 58 patients with adenomas, and 175 healthy individuals in the Chinese population. Yu et al. [34] observed high CRNDE-p expression and low miR-217 expression in the serum exosomes of patients with CRC compared to patients with adenoma or healthy individuals, according to qRT-PCR. The same study also demonstrated that high CRNDE-p expression and low miR-217 expression in the exosomes were related to advanced tumor stages (T3 and T4) and clinical stages (III and IV) of CRC, in addition to the presence of lymph node metastasis and distant metastasis. An AUC of 0.9326 for the combined expression of serum exosomal CRNDE-p and miR-217 suggested its potential as a diagnostic predictor compared to isolated individual factors and any conventional tumor marker (CRNDE-p, miR-217, and CEA alone or CRNDE-p and CEA combined). The authors also compared the exosomal levels of CRNDE-p and miR-217 in paired serum samples from 10 pre- and post-chemotherapy patients and found lower levels of CRNDE-p expression and higher levels of miR-217 in post-chemotherapy samples compared to in pre-chemotherapy samples. In the plasma samples of 369 patients with CRC and healthy individuals from the Chinese population, Liu et al. [3] performed a four-phase study (discovery, training, validation, and external validation) using a GEO dataset, which was subsequently validated using qRT-PCR. The authors found that the expression of exosomal miR-27a and miR-130a was higher in patients with CRC than in patients with adenoma and healthy individuals (AUC = 0.746 and 0.697, respectively) and was associated with TNM staging, histological grading, and lower OS. The authors also analyzed plasma samples from pre- and postoperative patients and found that the expression of exosomal miR-27a and miR-130a decreased significantly after surgical resection. The results presented in each phase of the study also demonstrated the diagnostic power of the combination of the two miRNAs (miR-27a and miR-130a), with an AUC of 0.801, a sensitivity of 80% and a specificity of 90% in patients with CRC. Tsukamoto et al. [35] evaluated the prognostic potential of miR-21 in the plasma exosomes of 326 Japanese patients with CRC according to the TNM stage (51 stage I, 110 stage II, 98 stage III, and 67 stage IV). Using microarray and qRT-PCR analysis, the authors found that significant levels of exosomal miR-21 expression in the plasma were related to a poor prognosis for OS and DFS in patients with TNM stage II and III CRC, and a poor OS only among TNM stage IV patients. The following year, Shao et al. [36] conducted a study with 30 patients with adenoma, 65 patients with CRC with liver metastasis, and 60 patients with CRC without liver metastasis, and 80 healthy individuals in China. Using tCLN biochip analysis (Tied Cationic Lipoplex Nanoparticles), the authors demonstrated an increase in the expression of exosomal miR-21 and IL-6 in the plasma of patients with metastatic CRC, suggesting that they are associated with the progression of CRC. 

Interestingly, tumor-draining veins have a greater potential to detect exosomal miRNAs retained in the target organ, such as the liver, than in the peripheral vein (PV). Accordingly, Santasusagna et al. [37] analyzed the miRNAs of the miR-200 family in the plasma of the mesenteric vein (MV) and PV of 50 patients with stage I–III colon cancer in Spain. The expression levels of all members of the miR-200 family were found to be higher in MV than in PV. However, no significant association was observed between the exosome load in PV and MV. In addition, patients with a low exosomal load of miR-200c and miR-141 in MV had a longer OS. 

### 2.3. Exosomal miRNAs for Surveillance and Follow-up

In the search for potential biomarkers for prognosis and tumor recurrence, Matsumura et al. [38] showed that the expression of the miR-17-92a cluster in the exosomal serum was higher in patients with recurrent CRC than in healthy individuals using two different sets of serum samples: 6 samples of patients with CRC used for microarray and 221 samples of patients with CRC used for qRT-PCR and 28 healthy individuals from Japan. The median overall survival time after resection was 4.64 years, and tumor recurrence was observed after an average interval of 4.82 years. The expression of six exosomal miRNAs (miR-19a, miR-19b, miR-23a, miR-92a, miR-320a, and miR-4437) was found to be upregulated and synchronized with the development of liver metastasis. Among them, the expression of miR-19a and miR-92a were significantly increased in patients with CRC compared to healthy individuals at each stage of cancer. In a later study, Liu et al. [39] evaluated 84 participants from a US population with stage II/III colon cancer after tumor resection and before adjuvant therapy for a period of 51 months. The authors found that 10 exosomal miRNAs were differentially expressed in the serum using RNA sequencing (RNA-Seq) and qRT-PCR. miR-4772-3p was significantly unregulated, and miR-4732-5p was upregulated in patients with recurrent CRC compared to patients with nonrecurrent CRC. The ROC curve showed that exosomal miR-4772-3p was associated with an increased risk of tumor recurrence (AUC = 0.72) and showed potential for use in the identification of patients with stage II and III colon cancer. To determine the clinical significance of Glypican-1 (GPC1) circulating in exosomes of patients with CRC, Li et al. [40] analyzed plasma samples from 85 patients with stage III CRC in the Chinese population before and after surgery and followed patients for up to 2 years after surgery. Using flow cytometry, the concentrations of circulating plasma exosomes positive for GPC1 were found to be significantly higher before and after surgery, including among patients who had a recurrence and were associated with lower patient survival. In addition, the negative expression of miR-96-5p and miR-149 observed before surgery, analyzed by qRT-PCR, was found to be correlated with an advanced TNM stage, short survival, poor prognosis, and relapse in patients with stage III CRC. As previously reported [37], there is a greater potential for finding biomarkers in veins that drain the tumor. In a study on the Spanish population, Monzo et al. [41] compared the expression of exosomal miRNAs extracted from PV plasma and MV in 50 patients with stage I to III colon cancer who underwent surgical resection. In qRT-PCR analysis, 13 miRNAs were found to be differentially expressed between patients with recurrent and nonrecurrent CRC. Of these, four exosomal miRNAs (let-7g, miR-15b, miR-155, and miR-328) were significantly expressed in the plasma and were associated with a shorter recurrence time. In addition, the concentration of exosomal miR-328 in the MV group was higher in patients who developed metastasis. Tumors, such as CRC, can be heterogeneous and have regions with hypoxia. Hypoxic cells secrete large amounts of exosomes, which can be used as circulating markers of locally advanced rectal cancer (LARC). Recently, Bjørnetrø et al. [42] used plasma samples from 24 patients with LARC in Norway to analyze the expression of exosomal miRNAs in relation to hypoxia using qRT-PCR. The authors found that low levels of circulating miR-486-5p and miR-181a-5p in hypoxic cell line exosomes were associated with invasive primary tumors and lymph node metastases, respectively, in patients with LARC. Interestingly, miR-30d-5p was found to be upregulated in patients with LARC with metastatic progression.

### 2.4. Exosomal miRNAs for the Management of Metastatic Disease 

Due to the importance of identifying predictive biomarkers for chemoresistance in patients with CRC, Jin et al. [43] analyzed serum samples from 43 patients with CRC stages III and IV before chemotherapy with 5-fluorouracil (5-FU), oxaliplatin, and leucovorin. The authors reported that a panel of four exosomal miRNAs (miR-21-5p, miR-96-5p, miR-1246, and miR-1229-5p) were capable of distinguishing between patients with chemosensitive and chemoresistant CRC, with an AUC of 0.804, a sensitivity of 78.00%, and a specificity of 88.90%. Recently, Yagi et al. [44] evaluated the expression of exosomal miRNAs associated with resistance to chemotherapy, namely mFOLFOX6, in the plasma of three patients with CRC and stable disease (SD), three with DFS, and three healthy individuals in the Japanese population, using microarray analysis, in addition to analyzing the expression of exosomal miR-125b in 55 patients with advanced/recurrent CRC by qRT-PCR. The results showed that exosomal miR-125b expression was significantly increased in patients with PD compared to patients with SD and healthy individuals and caused poor DFS. The authors suggested that exosomal miR-125b in the plasma is a potential biomarker of resistance to mFOLFOX6-based chemotherapy in patients with advanced or recurrent CRC.

## 3. Discussion

**Figure 1:**
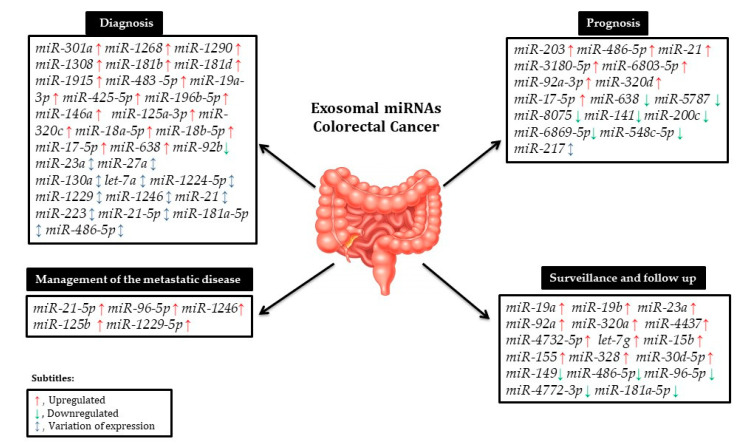
Circulating exosomal microRNAs (miRNAs) and their role in the diagnosis, prognosis, surveillance, and monitoring of colorectal cancer (CRC) metastatic disease.

As a result of emerging evidence, exosomes and their delivered miRNAs are regarded as potential new biomarkers of several types of cancer, including CRC [39]. The unique properties of exosomes, including their ability to incorporate specific miRNAs, their stability in circulation, their reproducible detection, and, most importantly, the fact that they reflect the properties of cancer cells [19], make them useful for early diagnosis and development of therapeutic strategies for patients with CRC (Figure 1). 

Twenty-eight experimental studies that described the association between exosomal miRNAs in CRC were included in this review. Among the 59 exosomal miRNAs suggested to be good candidates for use as CRC biomarkers (Supplementary Table S1), only two miRNAs (miR-6869-5p and miR-548c-5p) were downregulated in serum samples in more than one study [28,30,32]; these results were similar to those of liver metastasis and TNM stage in patients with CRC [28,30]. Reduced levels of miR-548c-5p in exosomes are associated with a poor prognosis in patients with CRC, regardless of whether the TNM stage is advanced, the tumor grade is low, and the liver has metastasized. As a result, it is a predictive factor for CRC prognosis. Thus, increasing evidence suggests that miR-548c-5p plays a role in cancer. However, few studies have addressed the presence of these miRNAs in circulating exosomes [45,46]. 

miR-21 has been shown to be positively regulated in patients with CRC [35,36] and is related to liver metastasis and TNM tumor staging in plasma samples. However, its expression was found to be reduced in serum samples after surgical resection of primary tumors [19]. Fukushima et al. [47] found that tissue samples from patients with CRC showed high levels of miR-21 expression and were associated with liver metastasis and Dukes stage. Therefore, further investigations are needed to elucidate the role of this miRNA in the prognosis of CRC.

Exosomal miR-486-5p was highly expressed in serum and plasma samples from patients with CRC [22,24]; however, its expression was reduced in the plasma samples of patients with primary organ tumors, in the postoperative period, with lymph node metastases [26,42]. Its expression was also reduced in the cell and tissue lines of patients with CRC. In addition, miR-486-5p was found to promote the proliferation and migration of CRC by activating the pleiomorphic adenoma gene-like 2 (PLAGL2), Insulin-like growth factor 2 (IGF2) andβ-catenin signaling pathway in vivo and in vitro, which is a promising therapeutic target for the treatment of patients with CRC [26]. Therefore, further studies are needed to demonstrate the role of exosomal miRNA expression patterns in vitro and in vivo in the activation pathways and circulation for use as prognostic factors in CRC.

miR-146a was positively expressed in the serum of patients with CRC and could be considered a diagnostic biomarker for the identification of the TME [22]. Although exosomes derived from cancer cells have been well characterized, few studies have explored the content and function of exosomes secreted by types of auxiliary cells found in the TME [48].

Although several technologies have been used (microarray, flow cytometry, and Tied Cationic Lipoplex Nanoparticles -tCLN) to obtain miRNA profiles, qRT-PCR remains the method of choice because of its high sensitivity and specificity [49]. It is worth noting that there is significant variability in the sample processing protocols and methods used for isolating exosomes and detecting miRNAs in the studies reviewed here (Table 1). The variability of miRNAs observed in all studies may be related to the different types of samples (e.g., plasma and serum), in addition to the intrinsic diversity of each population included in the studies. The heterogeneous results can be explained by the patient selection strategy (sample size, tumor, and clinical and pathological stages), sample collection and processing, and the different approaches for the analysis of exosomal miRNAs as well as the use of different cohort values for the same miRNAs [50]. In addition, methods need to be optimized to detect these specific circulating miRNAs for use in studies with a larger patient population. Interestingly, some authors chose not to elucidate all of the exosomal miRNAs that were expressed positively or negatively in patients with CRC, which may have led to inaccurate conclusions. Further research will be necessary to identify reliable circulating exosomal miRNAs for use as biomarkers for early diagnosis and prognosis of patients with CRC.

## 4. Materials and Methods

### 4.1. Bibliographic Search Strategy

We conducted a review of original articles from the current literature (2015 to 2019) using the bibliographic search engine of the US National Library of Medicine MEDLINE/PubMed (www.ncbi.nlm.nih.gov/pubmed). Keywords for the search included a combination of the following terms: “colorectal cancer exosome in blood”, “colorectal cancer exosome in plasma”, “serum exosome in colorectal cancer”, “circulating colorectal cancer exosome”, “microRNA,” “miRNA”, and “miR.”

### 4.2. Selection of Studies

**Figure 2:**
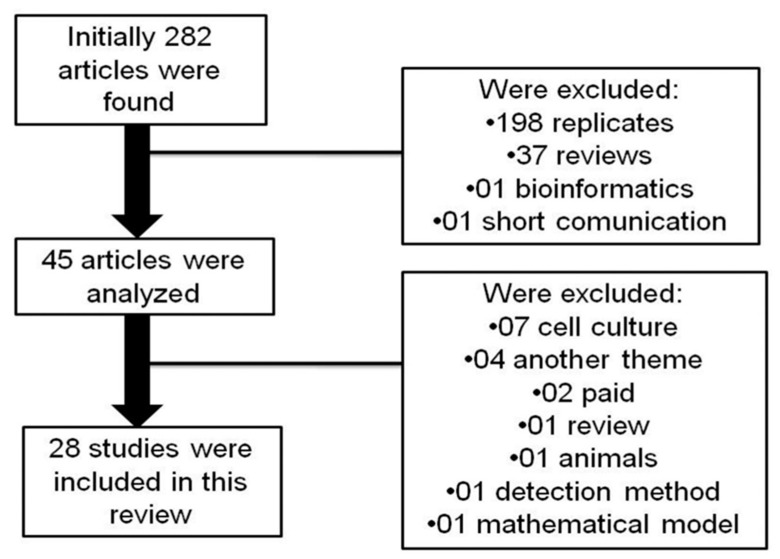
Flowchart of the study selection process.

The inclusion criteria were studies published in the last 5 years in English, selected according to the title and summary, which evaluated the potential of plasma/serum exosomal miRNAs in human cell lines for use as biomarkers for the diagnosis, prognosis, and pathogenicity of CRC. The exclusion criteria were studies using animal models, studies based on cell culture, brief communications, other reviews on the subject, or bioinformatics analyses. A total of 282 articles were detected in the preliminary search using 12 combinations of terms, of which the following were excluded: 198 were replicates, 39 did not meet the inclusion criteria, 37 were bibliographic reviews, one was a bioinformatics analysis, and one was a short communication. In the remaining 45 articles, 17 were excluded after screening the abstract of the articles because they included studies describing cell culture, paid articles, articles covering other topics, systematic reviews, animal models, detection methods, and mathematical models. Ultimately, 28 articles were selected for this review and met the inclusion criteria, including those involving ROC and AUC curve analysis (Figure 2). 

## 5. Conclusions

The role of circulating miRNAs in patients with CRC is attracting increasing attention. In fact, in the last 5 years, there has been a significant increase in the number of publications associating miRNAs with CRC (www.ncbi.nlm.nih.gov/pubmed). However, few studies have assessed this association with miRNAs packed in exosomes. Therefore, studies that look for potential targets of this condition for therapy and validate these exosomal miRNAs in larger sample cohorts should be encouraged. Several studies have identified associations between expressions of circulating exosomal miRNAs and the diagnosis, prognosis, and resistance to chemotherapy in patients with CRC. However, improving the specificity of these miRNA biomarkers remains a significant challenge. The use of biomarkers in conjunction with current screening methods may provide a path for their initial adoption in clinical practice. Therefore, this review is the starting point to explore the relevance of research in the search for exosomal circulating miRNAs that can be validated by in vivo and in vitro studies as biomarkers for CRC.

## Figures and Tables

**Table 1:** Review of sample protocols, isolation methods, detection and validation of exosomal miRNAs in the circulation.

Authors	Sample Type	Sample Processing Conditions	Method of Exosome Isolation	Method of RNA/miRNA Isolation	Method of miRNA Detection
Karimi et al., 2019	Serum	<2 hours of collection; 2000× *g* centrifugation for 10 min	ExoQuick (System Biosciences)	Not described	qRT-PCR
Liu et al., 2018	Plasma	Not described	Invitrogen Total Exosome Isolation Kit (Invitrogen)	miRNeasy Mini Kit (Qiagen)	qRT-PCR
Ogata-Kawata et al., 2014	Serum	Not described	Ultracentrifugation at 120,000× *g* for 70 min	Trizol LS reagent (Invitrogen)	Microarray and qRT-PCR
Zhu et al., 2017	Serum	Centrifugation at 1500 RPM for 10 min in 12 h after collection	ExoQuick Exosome Precipitation (System Biosciences)	mirVana Paris Kit ((Ambion)	qRT-PCR
Ren et al., 2017	Serum	Not described	exoRNeasy Serum/Plasma Max Kit	RNA Isolation Kit-miRNeasy Mini Kit	qRT-PCR
Cheng et al., 2019	Serum	Not described	Ultracentrifugation at 100,000× *g* for 120 min at 4 °C	Trizol reagent (Invitrogen)	qRT-PCR
Wang et al., 2017	Plasma	Centrifugation at 3000× *g* for 10 min 30 min after collection	ExoQuick Exosome Precipitation Solution (SBI)	miRNeasy Micro Kit (QIAGEN, Valencia, CA)	qRT-PCR
Zhang et al., 2019	Plasma	Centrifugation at 350 RCF for 10 min in 12 h	ExoQuick Exosome Precipitation Solution (System Biosciences)	mirVana Paris Kit (Ambion, Austin, TX, USA)	qRT-PCR
Min et al., 2019	Plasma	centrifugation at 3000× *g* for 15 min at 4°C	ultracentrifugation at 150,000× *g*, at 4 °C for 4 h	miRNeasy® Mini kit (Qiagen)	qRT-PCR
Liu et al., 2018	Plasma	Not described	Not described	Trizol LS Reagent (Invitrogen, CA, USA)	qRT-PCR
Takano et al., 2017	Serum	Centrifugation at 3000 RPM for 10 min at 4°C	Ultracentrifugation at 100,000× *g* for 70 min at 4 °C	miRNeasy serum/plasma kit (Qiagen)	qRT-CR
Yan et al., 2017	Serum	Centrifugation at 2000 RPM for 10 min	Total Exosome Isolation Kit (Invitrogen)	Trizol LS (Invitrogen) and miRNeasy mini kit (Qiagen)	Microarray and qRT-PCR
Yan et al., 2018	Serum	Centrifugation at 1200× *g* for 10 min at 4 °C	Invitrogen™ Total Exosome Isolation Kit	miRNeasy mini kit (Qiagen)	qRT-PCR
Yan et al., 2018	Serum	Not described	Total exosome isolation kit (Invitrogen)	Trizol reagent (Invitrogen)	qRT-PCR
Fu et al., 2018	Serum	Centrifugation at 1500 RPM for 10 min, and centrifugation at 12,000 RPM for 2 min within 12 h of collection	Exosome Isolation Reagent RiboTM	HiPure Liquid RNA/miRNA Kit (Magen, China)	qRT-PCR
Peng; Gu; Yan., 2019	Serum	Centrifugation at 1500–2000 RPM for 20 min	Total exosome isolation kit (Invitrogen)	Trizol reagent (Invitrogen miRNeasy mini kit (Qiagen)	qRT-CR
Tang et al., 2019	Serum	Centrifugation at 10,000× *g* at 4 °C for 30 min	Ultracentrifugation at 100,000× *g* for 120 min at 4 °C	Trizol reagent	qRT-PCR
Yu et al., 2017	Serum	Centrifugation at 3000× *g* for 15 min at 4 °C	ExoQuick Exosome Precipitation Solution (SBI)	MirVana microRNA isolation kit (Life technologies)	qRT-CR
Tsukamoto et al., 2017	Plasma	Centrifugation at 1200× *g* for 10 min at 4 °C	Ultracentrifugation at 15,000× *g* for 70 min at 4 °C	miRNeasy Serum/Plasma Kit (Qiagen, Hilden, Germany)	Microarray and qRT-PCR
Shao et al., 2018	Plasma	Not described	Ultracentrifugation at 160,000× *g* for 16 h	tCLN biochip	tCLN biochip
Santasusagna et al., 2018	Plasma	Centrifugation at 5000× *g* for 10 min	Ultracentrifugation	miRNeasy Mini Kit (Qiagen, Valencia, CA, USA)	qRT-PCR
Matsumura et al., 2015	Serum	Not described	Total Exosome Isolation Kit (Invitrogen)	miRNeasy mini kit (Qiagen)	Microarray and qRT-PCR
Liu et al., 2016	Serum	Centrifugation at 3000× *g* for 10 min at ambient temperature immediately after blood collection	ExoQuick kit (SBI)	SeraMir exosome RNA kit (SBI)	RNA-Seq and qRT-PCR
Li et al., 2017	Plasma	Centrifugation at 3000× *g* for 5 min at 4 °C	ExoCapTM Exosome Isolation and Enrichment kit (JSR)	Trizol reagent (Invitrogen, Carlsbad, CA, USA)	Flow cytometry test and qRT-PCR
Monzo et al., 2017	Plasma	Centrifugation at 5000× *g* for 10 min	Ultracentrifugation	miRNeasy Mini Kit (Qiagen, Valencia, CA, USA)	qRT-PCR
Bjørnetrø et al., 2019	Plasma	Centrifugation at 2000× *g* for 10 min	miRCURY™ Exosome Isolation Kit (Exiqon)	miRNeasy (Qiagen, Hilden, Germany)	qRT-PCR
Jin et al., 2019	Serum	Centrifugation at 2000 RPM for 10 min	Total Exosome Isolation Kit (Invitrogen)	Trizol Reagent (Invitrogen, Carlsbad, CA)	qRT-PCR
Yagi et al., 2019	Plasma	Centrifugation at 1200 × *g* for 10 min at 4 °C	Ultracentrifugation at 100,000× *g* for 70 min at 4 °C	miRNeasy serum/plasma kit (Qiagen, Hilden, Germany)	Microarray and qRT-PCR

qRT-PCR: real-time quantitative polymerase chain reaction; RPM: Rotations Per Minute; RCF: Relative Centrifugal Force; SBI: System Biosciences; RNA-Seq: RNA sequencing; tCLN: Tied Cationic Lipoplex Nanoparticles.

## Data Availability

The data that support the findings of this study are available from the corresponding author upon reasonable request.

## Supplementary Materials

Supplementary materials can be found at https://www.mdpi.com/1422-0067/22/1/346/s1.

## References

[1] Bray F., Ferlay J., Soerjomataram I., Siegel R.L., Torre L.A., Jemal A. (2018). Global cancer statistics 2018: GLOBOCAN estimates of incidence and mortality worldwide for 36 cancers in 185 countries. CA Cancer J. Clin..

[2] Karimi N., Feizi M.A.H., Safaralizadeh R., Hashemzadeh S., Baradaran B., Shokouhi B., Shahram T. (2019). Serum overexpression of miR-301a and miR-23a in patients with colorectal cancer. J. Chin. Med. Assoc..

[3] Liu X., Pan B., Sun L., Chen X., Zeng K., Hu X., Xu T., Xu M., Wang S. (2018). Circulating exosomal miR-27a and miR-130a act as novel diagnostic and prognostic biomarkers of colorectal cancer. Cancer Epidemiol. Biomark. Prev..

[4] Hibner G., Kimsa-Furdzik M., Francuz T. (2018). Relevance of MicroRNAs as Potential Diagnostic and Prognostic Markers in Colorectal Cancer. Int. J. Mol. Sci..

[5] Falzone L., Scola L., Zanghi A., Biondi A., Di Cataldo A., Libra M., Candido S. (2018). Integrated analysis of colorectal cancer microRNA datasets: Identification of microRNAs associated with tumor development. Aging (Albany NY).

[6] Weng W., Feng J., Qin H., Ma Y., Goel A. (2015). An update on miRNAs as biological and clinical determinants in colorectal cancer: A bench-to-bedside approach. Future Oncol..

[7] Xu P., Zhu Y., Sun B., Xiao Z. (2016). Colorectal cancer characterization and therapeutic target prediction based on microRNA expression profile. Sci. Rep..

[8] Ortiz-Quintero B. (2016). Cell-free microRNAs in blood and other body fluids, as cancer biomarkers. Cell Prolif..

[9] Gallo A., Tandon M., Alevizos I., Illei G.G. (2012). The Majority of MicroRNAs Detectable in Serum and Saliva Is Concentrated in Exosomes. Afarinkia, K., editor. PLoS ONE.

[10] Mitchell P.S., Parkin R.K., Kroh E.M., Fritz B.R., Wyman S.K., Pogosova-Agadjanyan E.L., Peterson A., Noteboom J., O’Briant K.C., Allen A. (2008). Circulating microRNAs as stable blood-based markers for cancer detection. Proc. Natl. Acad. Sci. USA.

[11] Khoury S., Tran N. (2015). Circulating microRNAs: Potential biomarkers for common malignancies. Biomarkers in Medicine.

[12] Fadaka A.O., Pretorius A., Klein A. (2019). Biomarkers for Stratification in Colorectal Cancer: MicroRNAs. Cancer Control.

[13] Balacescu O., Sur D., Cainap C., Visan S., Cruceriu D., Manzat-Saplacan R., Muresan M.S., Balacescu L., Lisencu C., Irimie A. (2018). The Impact of miRNA in Colorectal Cancer Progression and Its Liver Metastases. Int. J. Mol. Sci..

[14] Shirafkan N., Mansoori B., Mohammadi A., Shomali N., Ghasbi M., Baradaran B. (2018). MicroRNAs as novel biomarkers for colorectal cancer: New outlooks. Biomed. Pharmacother..

[15] Soung Y.H., Ford S., Zhang V., Chung J. (2017). Exosomes in cancer diagnostics. Cancers (Basel)..

[16] Lv J., Zhao H.P., Dai K., Cheng Y., Zhang J., Guo L. (2020). Circulating exosomal miRNAs as potential biomarkers for Barrett’s esophagus and esophageal adenocarcinoma. World J. Gastroent..

[17] Roberts C.T., Kurre P. (2013). Vesicle trafficking and RNA transfer add complexity and connectivity to Cell-cell communication. Cancer Res..

[18] Fang T., Lv H., Lv G., Li T., Wang C., Han Q., Wang H. (2018). Tumor-derived exosomal miR-1247-3p induces cancer-associated fibroblast activation to foster lung metastasis of liver cancer. Nat. Commun..

[19] Ogata-Kawata H., Izumiya M., Kurioka D., Honma Y., Yamada Y., Furuta K., Gunji T., Ohta H., Okamoto H., Sonoda H. (2014). Circulating Exosomal microRNAs as Biomarkers of Colon Cancer. Akagi, T., editor. PLoS ONE.

[20] Zhu M., Huang Z., Zhu D., Zhou X., Shan X., Qi L.W., Wu L., Cheng W., Zhu J., Zhang L. (2017). A panel of microRNA signature in serum for colorectal cancer diagnosis. Oncotarget.

[21] Ren D., Lin B., Zhang X., Peng Y., Ye Z., Ma Y., Liang Y., Cao L., Li X., Li R. (2017). Maintenance of cancer stemness by miR-196b-5p contributes to chemoresistance of colorectal cancer cells via activating STAT3 signaling pathway. Oncotarget.

[22] Cheng W., Liao T., Lin C., Yuan L.E., Lan H., Lin H., Teng W.H., Chang H.C., Lin C.H., Yang C.Y. (2019). RAB27B-activated secretion of stem-like tumor exosomes delivers the biomarker microRNA-146a-5p, which promotes tumorigenesis and associates with an immunosuppressive tumor microenvironment in colorectal cancer. Int. J. Cancer.

[23] Wang J., Yan F., Zhao Q., Zhan F., Wang R., Wang L., Zhang Y., Huang X. (2017). Circulating exosomal miR-125a-3p as a novel biomarker for early-stage colon cancer. Sci. Rep..

[24] Zhang H., Zhu M., Shan X., Zhou X., Wang T., Zhang J., Tao J., Cheng W., Chen G., Li J. (2019). A Panel of Seven-MiRNA Signature in Plasma as Potential Biomarker for Colorectal Cancer Diagnosis. Gene.

[25] Min L., Chen L., Liu S., Yu Y., Guo Q., Li P., Zhu S. (2019). Loss of Circulating Exosomal MiR-92b Is a Novel Biomarker of Colorectal Cancer at Early Stage. Int. J. Med. Sci..

[26] Liu X., Chen X., Zeng K., Xu M., He B., Pan Y., Sun H., Pan B., Xu X., Xu T. (2018). DNA-methylation-mediated silencing of miR-486-5p promotes colorectal cancer proliferation and migration through activation of PLAGL2/IGF2/β-catenin signal pathways. Cell Death Dis..

[27] Takano Y., Masuda T., Iinuma H., Yamaguchi R., Sato K., Tobo T., Hirata H., Kuroda Y., Nambara S., Hayashi N. (2017). Circulating exosomal microRNA-203 is associated with metastasis possibly via inducing tumor-associated macrophages in colorectal cancer. Oncotarget.

[28] Yan S., Han B., Gao S., Wang X., Wang Z., Wang F., Zhang J., Xu D., Sun B. (2017). Exosome-encapsulated microRNAs as circulating biomarkers for colorectal cancer. Oncotarget.

[29] Yan S., Jiang Y., Liang C., Cheng M., Jin C., Duan Q., Xu D., Yang L., Zhang X., Ren B. (2018). Exosomal miR-6803-5p as potential diagnostic and prognostic marker in colorectal cancer. J. Cell Biochem..

[30] Yan S., Liu G., Jin C., Wang Z., Duan Q., Xu J., Xu D. (2018). MicroRNA-6869-5p acts as a tumor suppressor via targeting TLR4/NF-κB signaling pathway in colorectal cancer. J. Cell. Physiol..

[31] Fu F., Jiang W., Zhou L., Chen Z. (2018). Circulating Exosomal MiR-17-5p and MiR-92a-3p Predict Pathologic Stage and Grade of Colorectal Cancer. Transl. Oncol..

[32] Peng Z., Gu R., Yan B. (2019). Downregulation of exosome-encapsulated miR-548c-5p is associated with poor prognosis in colorectal cancer. J. Cell. Biochem..

[33] Tang Y., Zhao Y., Song X., Song X., Niu L., Xie L. (2019). Tumor-derived exosomal miRNA-320d as a biomarker for metastatic colorectal cancer. J. Clin. Lab. Anal..

[34] Yu B., Du Q., Li H., Liu H.Y., Ye X., Zhu B., Zhai Q., Li X.X. (2017). Diagnostic Potential of Serum Exosomal Colorectal Neoplasia Differentially Expressed Long Non-Coding RNA (CRNDE-p) and MicroRNA-217 Expression in Colorectal Carcinoma. Oncotarget.

[35] Tsukamoto M., Iinuma H., Yagi T., Matsuda K., Hashiguchi Y. (2017). Circulating Exosomal MicroRNA-21 as a Biomarker in Each Tumor Stage of Colorectal Cancer. Oncology.

[36] Shao Y., Chen T., Zheng X., Yang S., Xu K., Chen X., Xu F., Wang L., Shen Y., Wang T. (2018). Colorectal cancer-derived small extracellular vesicles establish an inflammatory premetastatic niche in liver metastasis. Carcinogenesis.

[37] Santasusagna S., Moreno I., Navarro A., Rodenas F.M., Hernández R., Castellano J.J., Muñoz C., Monzo M. (2018). Prognostic impact of miR-200 family members in plasma and exosomes from tumor-draining versus peripheral veins of colon cancer patients. Oncology.

[38] Matsumura T., Sugimachi K., Iinuma H., Takahashi Y., Kurashige J., Sawada G., Ueda M., Uchi R., Ueo H., Takano Y. (2015). Exosomal microRNA in serum is a novel biomarker of recurrence in human colorectal cancer. Br. J. Cancer.

[39] Liu C., Eng C., Shen J., Lu Y., Yoko T., Mehdizadeh A., Chang G.J., Rodriguez-Bigas M.A., Li Y., Chang P. (2016). Serum exosomal miR-4772-3p is a predictor of tumor recurrence in stage II and III colon cancer. Oncotarget.

[40] Li J., Li B., Ren C., Chen Y., Guo X., Zhou L., Peng Z., Tang Y., Chen Y., Liu W. (2017). The clinical significance of circulating GPC1 positive exosomes and its regulative miRNAs in colon cancer patients. Oncotarget.

[41] Mariano M., Santasusagna S., Moreno I., Martinez F., Hernández R., Muñoz C., Castellano J.J., Moreno J., Navarro A. (2017). Exosomal MicroRNAs Isolated from Plasma of Mesenteric Veins Linked to Liver Metastases in Resected Patients with Colon Cancer. Oncotarget.

[42] Bjørnetrø T., Redalen K.R., Meltzer S., Thusyanthan N.S., Samiappan R., Jegerschöld C., Handeland K.R., Ree A.H. (2019). An experimental strategy unveiling exosomal microRNAs 486-5p, 181a-5p and 30d-5p from hypoxic tumour cells as circulating indicators of high-risk rectal cancer. J. Extracell. Vesicles.

[43] Jin G., Liu Y., Zhang J., Bian Z., Yao S., Fei B., Zhou L., Yin Y., Huang Z. (2019). A panel of serum exosomal microRNAs as predictive markers for chemoresistance in advanced colorectal cancer. Cancer Chemother. Pharmacol..

[44] Yagi T., Iinuma H., Hayama T., Matsuda K., Nozawa K., Tsukamoto M., Shimada R., Akahane T., Tsuchiya T., Ozawa T. (2019). Plasma exosomal microRNA-125b as a monitoring biomarker of resistance to mFOLFOX6-based chemotherapy in advanced and recurrent colorectal cancer patients. Mol. Clin. Oncol..

[45] Ge J., Li J., Na S., Wang P., Zhao G., Zhang X. (2019). miR-548c-5p inhibits colorectal cancer cell proliferation by targeting PGK1. J. Cell. Physiol..

[46] Ines B., Burton M., Sørensen K.P., Andersen L., Larsen M.J., Bak M., Cold S., Thomassen M., Tan Q., Kruse T.A. (2018). Association of MiR-548c-5p, MiR-7-5p, MiR-210-3p, MiR-128-3p with Recurrence in Systemically Untreated Breast Cancer. Oncotarget.

[47] Fukushima Y., Iinuma H., Tsukamoto M., Matsuda K., Hashiguchi Y. (2015). Clinical significance of microRNA-21 as a biomarker in each Dukes’ stage of colorectal cancer. Oncol. Rep..

[48] McCann J.V., Liu A., Musante L., Erdbrügger U., Lannigan J., Dudley A.C. (2019). A miRNA signature in endothelial cell-derived extracellular vesicles in tumor-bearing mice. Sci. Rep..

[49] Danese E., Minicozzi A.M., Benati M., Paviati E., Gusella M. (2017). Reference MiRNAs for Colorectal Cancer: Analysis and Verification of Current Data. Sci. Rep..

[50] Rapado-González Ó., Majem B., Álvarez-Castro A., Díaz-Peña R., Abalo A., Suárez-Cabrera L., Gil-Moreno A., Santamaría A., López-López R., Muinelo-Romay L. (2019). A Novel Saliva-Based miRNA Signature for Colorectal Cancer Diagnosis. J. Clin. Med..

